# Causality in statistics and data science education

**DOI:** 10.1007/s11943-022-00311-9

**Published:** 2022-11-09

**Authors:** Kevin Cummiskey, Karsten Lübke

**Affiliations:** 1 Department of Mathematical Sciences, United States Military Academy, 10996 West Point, NY USA; 2 ifes Institute for Empirical Research & Statistics, FOM University of Applied Sciences, Dortmund, Germany

**Keywords:** Statistics education research, Data Science, Causality, Bias and Confounding, A22, C18, C55, C80, C90

## Abstract

Statisticians and data scientists transform raw data into understanding and insight. Ideally, these insights empower people to act and make better decisions. However, data is often misleading especially when trying to draw conclusions about causality (for example, Simpson’s paradox). Therefore, developing causal thinking in undergraduate statistics and data science programs is important. However, there is very little guidance in the education literature about what topics and learning outcomes, specific to causality, are most important. In this paper, we propose a causality curriculum for undergraduate statistics and data science programs. Students should be able to think causally, which is defined as a broad pattern of thinking that enables individuals to appropriately assess claims of causality based upon statistical evidence. They should understand how the data generating process affects their conclusions and how to incorporate knowledge from subject matter experts in areas of application. Important topics in causality for the undergraduate curriculum include the potential outcomes framework and counterfactuals, measures of association versus causal effects, confounding, causal diagrams, and methods for estimating causal effects.

## Introduction

### Motivation

Friedrich et al. (2021), based on the position paper ‘DAGStat Stellungnahme: Die Rolle der Statistik in der Künstlichen Intelligenz’, ask: “Is there a role for statistics in artificial intelligence?”. They argue that statistics in times of artificial intelligence is important not only for design, data quality, and uncertainty assessment, but also for the differentiation between causality and associations. The ability to answer causal questions is not only important for artificial intelligence but also for many other data use cases.

Modern data scientists, statisticians, and other analysts rarely perform formal inference in the context of traditional randomized controlled trials and other experimental designs. Quite often, they assess causality while exploring complex data in observational settings. Understanding causality beyond the traditional randomized controlled trial is essential (Hernán et al. 2019; Bojinov et al. 2020). Gelman and Vehtari (2021) list causal inference as one of the most important statistical ideas of the past 50 years. Recent guidelines recommend students in statistics and undergraduate data science programs understand causality (GAISE 2016; De Veaux et al. 2017; NASEM 2018; ACM 2021) and there are several papers discussing ways to integrate causality into undergraduate statistics courses (Ridgway 2016; Kaplan 2018; Cummiskey et al. 2020; Lübke et al. 2020; Forney and Mueller 2021). Utts (2021) places the topics of “observational studies, confounding and causation” in her list of important topics for statistical literacy.

Integrating causality helps statistics and data science students develop a framework to think about the data generating process and to think more clearly about modeling and interpretation of results (Cummiskey et al., 2020; Lübke et al. 2020). In addition, it more closely aligns their education with the proposals of De Veaux et al. (2017) and Donoho (2017) for data science programs. However, there is limited discussion in the literature of how students, with regards to causality, should be able to think and what they should be able to do after completing their studies. Causal inference is a huge field of knowledge spanning several disciplines (statistics, economics, computer science, epidemiology, etc) and employing numerous methods; undergraduate programs cannot cover them all and must choose carefully. Further complicating this choice is the fact that causal inference, due to its recent emergence as a subfield of statistics, was not part of the formal education of many curriculum designers. The goal of this paper is to recommend to curriculum designers of undergraduate programs learning outcomes and a small set of important concepts for developing causal thinking. We intend the learning outcomes and concepts to apply to most programs while leaving flexibility in the methods and applications.

This paper is organized as follows. First, we discuss causal thinking and its connection to data literacy. Next, we highlight some key ideas in causal inference which provide students with formal definitions and rules to help structure causal thinking. Finally, we recommend learning outcomes and key concepts for undergraduate data science programs.

### Causal thinking

In terms of causality, the central goal of undergraduate data science and statistics programs should be to develop causal thinking in students. Causal thinking is a broad pattern of thinking that enables individuals to appropriately assess claims of causality based upon statistical evidence. Causal thinkers are naturally skeptical of causality claims from observational studies and recognize the human tendency to develop causal links between events even when none exist. At the same time, they have a richer understanding of causality than simply “correlation does not imply causation” and appreciate that we can, and often should, draw causal conclusions from observational studies. For example, there has never been a randomized controlled trial linking smoking with lung cancer, but overwhelming evidence from observational studies provides compelling evidence. Rohrer et al. (2021) stated “the only thing that can stop bad causal inference is good causal inference.” Humans will continue to make causal conclusions from data; it is our responsibility as educators to make our students better at it.

Causal thinking underpins many aspects of data literacy. The idea of data literacy augments the traditional ideas of statistical literacy (critically consuming statistics produced by others) with other important parts of the life cycle of data and how data is used in modern society (Gould 2017). Schüller (2020) defines data literacy as the cluster of all efficient behaviors and attitudes for creating value or making decisions from data. The final step in this process of creating value is the competence of deriving action from data. Often, this means asking “what if” questions about interventions that change some aspect of the system. For example, researchers investigating the effects of school mask mandates on COVID-19 infections might ask, “what if a school without a mandate implemented one?” This question is complicated by the fact that schools with mask mandates are likely different in ways that are important to infection than schools without mask mandates. Ideas in causal inference provide us with ways to think about these concepts.

### Causal inference

In this section, we discuss some important ideas in causal inference that help students develop causal thinking. One goal of causal inference is to estimate the effect of an intervention or exposure on an outcome of interest. Causal effects compare the outcome in the population under the intervention to the outcome without the intervention. The central challenge in estimating causal effects is, typically, we cannot observe the same individual both with and without the intervention. Randomizing the intervention allows for unbiased estimates of causal effects as individuals in both groups are similar in expectation assuming full adherence. However, randomized designs are not always feasible, ethical, or desirable and researchers often perform observational studies with the goal of estimating causal effects. (Even in experimental designs, practical issues often impede full adherence to the planned experimental design making it necessary to adopt causal inference approaches.) In observational studies, measures of association relating the intervention to the outcome are typically biased estimates of causal effects due to confounding. Confounding occurs when subjects who received the intervention are different from subjects who did not receive the intervention in ways that are themselves causes of the outcome.

For example, if we conducted a survey shortly after the release of the COVID-19 vaccine and compare all-cause mortality rates in individuals who receive the vaccine (the intervention, in this example) to individuals who did not receive the vaccine, we will likely find an association between receiving the vaccine and *higher* all-cause mortality rates. This result could lead people to falsely believe vaccines increase mortality risk. However, at the time, governments were vaccinating the most at-risk individuals first. The vaccinated individuals were fundamentally different from the unvaccinated individuals in ways that are important causes of mortality (for example, age). In statistical terms, we would say the association we observed between vaccination and higher all-cause mortality is a biased estimate of the causal effect. At the beginning of the vaccination campaign, older individuals were more likely to be vaccinated and, due to the age, at higher mortality risk—and not because of the vaccine. In other words, the higher mortality in the vaccinated group is caused by the higher age of the vaccinated group, not by the vaccine. In observational studies, when researchers measure a sufficient set of confounding variables, i.e. variables that influence both the exposure and the outcome, they can obtain unbiased estimates of causal effects using a variety of designs and statistical methods under the assumption of a correct causal model. In the previous example, age is a confounding variable for effect of vaccination status on mortality. Researchers identify confounding variables using subject matter knowledge to develop causal models; it is not possible to identify causal models and unmeasured confounding variables from the data alone. In the vaccine example above, it is not possible to detect confounding with only vaccination and mortality data. Researchers had to understand the data collection process and other mortality factors to identify age as an important confounding variable.

Causal diagrams are useful tools for identifying confounding variables and depicting causal models. Following simple heuristics for causal diagrams, researchers can determine a sufficient set of variables to control for to obtain unbiased estimates of causal effects (see e.g. Peters et al. 2017, p. 109ff). Importantly, the correctness of the causal inference depends on the correctness of the causal diagram. Therefore, subject matter knowledge and care are needed to develop the causal model.

## Causality in the undergraduate curriculum

### The undergraduate curriculum

Undergraduate statistics and data science programs should prepare students for a data-rich and complex world. Students should master fundamental concepts in statistics, computer science, and mathematics while gaining experience with all steps of the data cycle. De Veaux et al. (2017) describe six key competencies of an undergraduate data science major. They are computational and statistical thinking, mathematical foundations, model building and assessment, algorithms and software foundation, data curation, and knowledge transference. All courses should include significant data experiences where students must obtain, wrangle, and prepare data for analysis. Donoho (2017) divides the activities of data science into six divisions: data gathering, preparation, exploration; data representation and transformation, computing with data, data modeling, data visualization and presentation, and science about data science. Statistics and machine learning courses tend to focus on data modeling with little formal education in the others, especially data gathering, preparation, and exploration which takes up most of practitioners’ time. In a survey of introductory data sciences courses, Schwab-McCoy et al. (2021) report visualization, cleaning, and management, as well as professional practices and (statistical) modeling are the top five most common topics.

### Causality learning outcomes

We propose the following causality-related learning outcomes for statistics and data science programs. Undergraduate statistics and data science students should:

- Be able to define causal effects and understand how they differ from associations.
- Understand the concept of a data generating process and assumptions necessary for drawing causal conclusions.
- Be able to identify common sources of bias and confounding in data and depict them in a causal diagram.
- Be able to apply design or adjustment-based methods to estimate causal effects.

### Causality topics

We recommend the following topics in causality for undergraduate statistics and data science majors with an emphasis on developing causal thinking through the investigation of cause-and-effect type questions with real-world data.

**Fig. 1:**
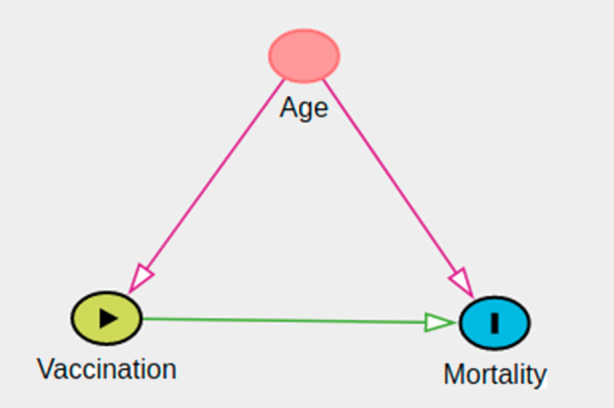
Causal diagram for the Covid example. The effect of Vaccination on Mortality is confounded by Age

- Potential Outcomes Framework—The potential outcomes framework provides undergraduate students with formal definitions of causality, mathematical notation for expressing causal effects, and common assumptions made when assessing causality. The definition of causality in statistics differs from its common usage in the English language and in other disciplines. For example, we might say pushing a door causes it to close. If I push the door with enough force, it will close every time (all else being equal). However, in statistics, we do not require this deterministic relationship between cause and effect. For example, we say smoking causes lung cancer even though not everyone who smokes will get lung cancer. Potential outcomes framework identifies the causal effect as the difference between two potential outcomes, the outcome if the individual smoked, *Y*_*1*_, and the outcome if the individual did not smoke, *Y*_*0*_, when averaged over the entire population. Traditional conditional probability notation cannot distinguish between causal effects and confounding. Let *A* be an indicator an individual smokes and *Y* be an indicator of lung cancer. *E(Y|A* *=* *1)* *−* *E(Y|A* *=* *0)* > 0 could either indicate smokers are more likely to get lung cancer (causality), or the smokers and nonsmokers are different in ways that cause lung cancer (confounding).
- Causal Effects and Confounding—Undergraduate students should reflexively think about confounding when presented with measures of association relating an intervention to an outcome. For example, a long running radio advertisement for a hospital located in the wealthiest county in New York touted that they had the shortest surgery recovery times in the entire state. Students should recognize some of the challenges to attributing shorter recovery times to the hospital (through better procedures, equipment, and expertise) instead of other factors (types of surgeries performed, baseline health of patients).
- Causal Diagrams—Causal diagrams are tools researchers use to depict the causal relationships between variables. The nodes in the graph are variables and an arrow (i.e. a directed edge) between them indicated a causal relationship. In many cases, using simple heuristics, researchers can determine a sufficient set of variables to control for during design and analysis to eliminate or reduce bias. For students, they are a valuable tool for structuring multivariable thinking and connecting contextual knowledge to statistical modeling decisions. It should be remembered that the correctness of the causal conclusion depends on the correctness of the causal model. With causal diagrams, modelling can be supported by qualitative background knowledge about the data generating process. As these assumptions are encoded in the diagram, communication and discussion of the results with other stakeholders is facilitated if the goal of the analysis is causal inference. Fig. 1 shows the assumed causal diagram of the COVID example. The diagram was made using DAGitty (Textor et al. 2016).
- Methods for Adjustment—students should gain experience with several basic methods for confounding adjustment. At a minimum, we recommend regression and matching. Regression provides an important opportunity to show students how the same method can be employed towards different objectives like prediction or causal inference. For example, when the goal of a study is prediction, we might automate variable selection to produce a parsimonious model that does well at predicting an outcome close to what we observe. However, when the goal of the study is to estimate a causal effect, it would be more appropriate to select variables to control for based on a presupposed causal diagram to control for confounding (exposure ← confounder → outcome), but not for mediators (exposure → mediator → outcome) and colliders (exposure → collider ← outcome). In the COVID vaccine example, we would control for Age by including it in the regression model because Age is confounding. However, if there was a mediator variable *M* of the Vaccination and Mortality relationship, we would not want to control for *M*, even if doing so improved the predictive performance of the model. Controlling for *M* results in a biased estimate of the causal effect. This is a different approach to teaching regression which typically focuses on improving the predictive performance and not estimating effects. Matching is also a very effective method for introducing students to causal thinking because the analysis after matching resembles that of a randomized treatment, thus reinforcing the notion of causality and common obstacles to obtaining good estimates of it.
- Design based approaches—in addition to classical randomized controlled trials, students should be aware of other designs such as natural experiments, instrumental variables, and regression discontinuity designs. Here we assume that most programs already include randomized experiments. If not, the topic of experimental design and the benefits of “randomness” within the data collection process should be discussed. Causal diagrams are an effective way to depict these designs. For example, a randomized experiment removes the arrow between the explanatory variable and the confounder.

### Resources

Statistics and data science are not the only disciplines with causality. Epidemiology, biostatistics, political sciences, economics, and computer science investigate research questions concerning causality. In the last years, several textbooks were published which may serve as the basic reading within data science courses addressing causality. We briefly highlight a few of them here.

*The book of why: the new science of cause and effect* by Pearl and Mackenzie (2018) introduces the topic for general audiences. In particular, the ladder of causality provides a nice framework for thinking about types of research questions and the limitations of algorithms to answer causality questions without human intervention. Aronow and Sävje 2020 provide some important critical notes in their review of the book. For a more mathematical treatment,* Causal inference in statistics: A primer*, by Pearl et al. (2016) covers many topics and provides a gentle introduction to causal inference. The ASA causality award winning book by Peters et al. (2017) *Elements of causal inference: foundations and learning algorithms* is written with a focus towards machine learning and may be more suitable for graduate courses. *Introduction to causal inference from a machine learning perspective *(Neal 2020) is currently still under development but provides additional resources like slides and videos. Alves (2020) C*ausal inference for the brave and true* integrates Python examples throughout. Hardt and Recht (2021) include causality in their P*atterns, predictions, and actions: A story about machine learning* book. Other textbooks that should be considered are Cunningham (2021), Hernán and Robins (2020) and Huntington-Klein (2021).

There are also several high-quality online courses. These are especially helpful for instructors for whom causal inference was not part of their formal education but want to integrate these topics into their existing courses. Harvard University offers the online course “Draw your assumptions before your conclusions” by Hernán on causal diagrams. The University of Pennsylvania offers “A Crash Course in Causality: Inferring Causal Effects from Observational Data” taught by Jason Roy, recent winner of the ASA’s Causality in Statistics Education Award. Columbia University offers a two-course series on causal inference taught by Michael Sobel. Udemy offers the course “Causal Data Science with Directed Acyclic Graphs” by Paul Hünermund.

## Discussion

The compelling progress of computational technology and the wide availability of diverse data makes the 21st century an exciting time for expanding opportunities in statistics and data science. We can analyze data in ways unpredictable when the foundations of statistical theory were developed 100 years ago. Despite this “data revolution” (Ridgway 2016), we should not forget the epistemological goals of an analysis. For communication of data-based results, it seems crucial to help students to think clearly about the assumptions of the data generating process to draw correct conclusions. The proposed undergraduate causality curriculum aims at providing a modeling framework to do so.

The causality topics discussed in this paper can be covered in a single course and only very few preliminaries are needed. Alternatively, they could be integrated in existing courses such as intermediate applied statistics, modeling, data science, regression, and generalized linear models. Some introductory textbooks are available, e.g. Pearl et al. (2016), as well as R or Python software for causal inference. Moreover, the concepts can be linked to other important aspects of modern statistics and data science and, therefore, can broaden the view on data and science.

Big data most often is multivariate observational data. Statisticians know about the pitfalls in drawing conclusions without random sampling or allocation. However, instead of just lamenting, we should explain the benefits of randomizing (erasing arrows in a causal diagram) and discuss the extra qualitative assumptions needed for causal inference. So our answer on Friedrich et al. (2021): “Is there a role for statistics in artificial intelligence?” is also yes. However, we need to adopt a richer understanding of causality in the curriculum.

Lastly, it should be noted that, to the best of our knowledge, there is limited empirical knowledge about the achieved learning outcomes. After implementation, students reasoning about correlation and causation, as well as the different tasks description such as prediction and causal inference, should be assessed.
